# The Hidden Burden: Perceived Stress Levels Among Chronic Obstructive Pulmonary Disease Patients and Its Correlation With Pulmonary Function

**DOI:** 10.7759/cureus.83358

**Published:** 2025-05-02

**Authors:** Noor N Rajpoot, Faran Maqbool, Noman Sadiq, Muhammad Tahir, Hazar Khan, Mukhtiar Ahmed

**Affiliations:** 1 Department of Medicine, Teaching Hospital, Mekran Medical College, Turbat, PAK; 2 Department of Medicine, Rawalpindi Medical University, Rawalpindi, PAK; 3 Department of Physiology, Mekran Medical College, Turbat, PAK; 4 Department of Pharmacology and Therapeutics, Mekran Medical College, Turbat, PAK; 5 Department of Pharmacology, Mekran Medical College, Turbat, PAK; 6 Department of Pathology, Jhalawan Medical College, Khuzdar, PAK

**Keywords:** chronic obstructive pulmonary disease, gender-differences, perceived stress score, psychological assessment, pulmonary function test

## Abstract

Background: Respiratory conditions such as chronic obstructive pulmonary disease (COPD) extend beyond physical symptoms to affect mental health. Current medical literature lacks a thorough exploration of psychological burdens in COPD, particularly regarding variations across different demographic populations.

Objective: This study sought to determine stress prevalence in individuals diagnosed with COPD and the relationship of perceived stress with the pulmonary function of the patients.

Methods: From December 2021 to May 2022, a cross-sectional study was conducted at Rawalpindi Teaching Hospital. A total of 100 clinically stable COPD patients were included in the study using a purposive sampling technique. Subjects' socio-demographic data were recorded, and asked to complete the standardized Perceived Stress Scale-10 (PSS-10) questionnaire. Data underwent descriptive statistics, including frequencies and mean calculations and correlation calculations for relationship assessment.

Results: The mean Perceived Stress Scale score was 18.38 ± 7.41 across all subjects (predominantly male, 89 (89%), mean age 53.88 ± 10.77). Among 100 COPD subjects, 26 (26%) were experiencing minimal stress (PSS 0-13), 59 (59%) moderate stress (PSS 14-26), and 15 (15%) severe stress (PSS 27-40). Most notably, female subjects demonstrated markedly elevated stress (22.64 ± 5.36) compared to their male counterparts (17.85 ± 7.46), reaching statistical significance (p=0.04). Inverse relationships emerged between pulmonary function measurements and stress intensity (vital capacity r=-0.19; forced vital capacity r=-0.16).

Conclusion: Our study identifies a substantial psychological burden among COPD patients, with nearly three-quarters experiencing moderate-to-severe stress and significant gender-based disparities. The findings of our study suggest that comprehensive COPD management should incorporate routine psychological evaluation with particular attention given to female patients.

## Introduction

Chronic obstructive pulmonary disease (COPD) represents a substantial global health challenge characterized by progressive airflow restriction stemming from combined small airway pathology and alveolar destruction [1]. COPD affects approximately 210 million individuals globally, and projections indicate it will become the third leading mortality cause worldwide by 2030. Beyond its immediate respiratory consequences, COPD creates a multifaceted healthcare burden encompassing both physiological manifestations and psychological impacts [2].

The cardinal manifestations of COPD, including dyspnea, persistent cough, and exercise intolerance, significantly compromise the quality of life [3]. However, the psychological dimensions frequently receive insufficient recognition in clinical contexts. Growing research suggests COPD patients encounter considerable emotional distress, including anxiety, depression, and heightened stress perception [4]. These psychological comorbidities can undermine therapeutic adherence, compromise management strategies, and reduce the effectiveness of smoking cessation initiatives crucial for COPD control [5].

Perceived stress refers to an individual’s subjective appraisal regarding life circumstances perceived as overwhelming [6]. For individuals managing chronic respiratory conditions like COPD, the ongoing challenges of breathing difficulty, functional limitations, and disease progression fears can substantially elevate perceived stress [7]. This emotional burden potentially varies across demographic categories, with possible differences based on gender, age distribution, and disease severity [8]. Perceived stress can be measured using the Perceived Stress Scale-10 (PSS-10), a widely validated 10-item questionnaire. The PSS-10 is a widely validated 10-item questionnaire that measures how unpredictable, uncontrollable, and overloaded respondents find their lives. PSS-10 captures subjective stress experiences over the past month rather than specific stressful events. The PSS-10 uses a simple scoring system (0-4 per item) to provide a total perceived stress score, making it one of the most widely used psychological instruments for measuring stress perception across diverse populations [9].

Despite the increasing acknowledgement of psychological components in COPD, focused research examining stress perception, specifically among these patients, remains insufficient, particularly in developing regions [10]. Furthermore, a notable knowledge gap exists regarding gender-specific variations in psychological responses to COPD, which could inform tailored intervention approaches [11].

This study aims to address these research gaps by assessing perceived stress prevalence among Pakistani COPD patients, with specific attention to gender-based differences and its correlation with pulmonary function tests. Understanding the psychological burden associated with COPD proves essential for developing holistic management strategies addressing both the physiological and psychological aspects of this complex medical condition.

## Materials and methods

Study design and ethical approval

This observational cross-sectional study was conducted at the Medicine Department at Rawalpindi Teaching Hospital from December 2021 through May 2022. The research received approval from Rawalpindi Medical University’s Institutional Review Board (M-25/19/RMU) and adhered to the ethical principles outlined in the Helsinki Declaration.

Study participants

Using a purposive convenience sampling methodology, we recruited 100 individuals with a confirmed COPD diagnosis. COPD diagnosis followed the Global Initiative for Chronic Obstructive Lung Disease criteria, considering clinical presentation, risk factor exposure, and spirometric confirmation of airflow limitation (post-bronchodilator forced expiratory volume in the first second (FEV1)/forced vital capacity (FVC) < 0.70) [12].

Inclusion criteria

Adults aged 35 years or older with a confirmed COPD diagnosis, clinical stability (no exacerbation events within a preceding month), and cognitive capacity to comprehend and complete assessment instruments.

Exclusion criteria

Drug abuse, concurrent significant respiratory conditions (bronchiectasis, interstitial disease), pre-existing psychiatric diagnosis before COPD development, and cognitive limitations affecting informed consent capacity or questionnaire completion.

Data collection

Following written informed consent, researchers gathered demographic information and clinical data using a structured proforma. Pulmonary function assessment, including vital capacity, FVC, and FEV1/FVC ratio measurements, employed spirometry following American Thoracic Society/European Respiratory Society technical standards [13].

Psychological assessment

We employed the validated 10-item Perceived Stress Scale to evaluate perceived stress. This widely recognized instrument measures the degree to which life situations are appraised as stressful [9]. The questionnaire explores how unpredictable, uncontrollable, and overwhelming respondents find their circumstances. Participants indicate the frequency of specific thoughts or feelings during the previous month using a 5-point scale (0=never to 4=very often).

Cumulative scores range from 0 to 40, with higher values indicating greater perceived stress. Based on established thresholds, participants were classified as experiencing low stress (scores 0-13), moderate stress (scores 14-26), or high stress (scores 27-40) [14].

Data analysis

Data analysis was done using SPSS version 25.0 (IBM Corp., Armonk, NY, USA). Descriptive statistics represented continuous variables as means with standard deviations and categorical variables as frequencies with percentages. The Shapiro-Wilk procedure assessed distribution normality. Gender-based differences in perceived stress were evaluated using an independent samples t-test. Pearson correlation coefficients examined the correlation between perceived stress scores and clinical parameters. The statistical significance threshold was established at p<0.05.

## Results

Demographic and clinical characteristics

The participant cohort included 100 COPD patients with a mean age of 53.88 ± 10.77 years. Male participants constituted the majority (89, 89%) of the sample. The mean Body Mass Index was 24.12 ± 3.50 kg/m² (range: 16.40-33.80), indicating most participants fell within the normal to overweight classification. Table 1 presents comprehensive demographic and clinical characteristics of study participants.

**Table 1:** Demographic and clinical characteristics of study participants The data are presented as either Mean ± SD or as %. FEV1: Forced expiratory volume in 1 second, FVC: Forced vital capacity

Parameter	Mean ± SD or n (%)
Age (years)	53.88 ± 10.77
Gender	
Male	89 (89%)
Female	11 (11%)
Body mass index (kg/m²)	24.12 ± 3.50
Pulmonary function parameters	
Vital capacity (L)	3.56 ± 0.43
Forced vital capacity (L)	3.50 ± 0.53
FEV1/FVC ratio (%)	71.27 ± 6.06

Prevalence of perceived stress

Assessment revealed a mean Perceived Stress Scale score of 18.38 ± 7.41 across all participants, indicating moderate stress levels predominated within the sample. Classification based on established thresholds showed that 26 (26%) participants experienced low stress levels, 59 (59%) participants exhibited moderate stress, and 15 (15%) participants demonstrated high stress levels.

Gender differences in perceived stress

Analysis of stress perception across gender groups revealed significant variations. Female participants exhibited a mean PSS score of 22.64 ± 5.36, substantially higher than the mean score of 17.85 ± 7.46 observed among male participants (p = 0.04). This finding indicates that female COPD patients experienced markedly more significant distress compared to their male counterparts.

Relationship between perceived stress and clinical parameters

Correlation analysis examining associations between perceived stress and clinical measurements revealed negative relationships. Results showed a weak negative correlation between vital capacity and perceived stress levels (r = -0.1912) and between FVC and perceived stress levels (r = -0.1553). These findings suggest that individuals with more compromised pulmonary function may experience higher perceived stress, although the correlation strength remained modest. No significant correlations emerged between perceived stress scores and FEV1/FVC ratio (r = 0.0597). Correlation of perceived stress with age showed no significant relationship (r = -0.011, p = 0.913), and similarly, perceived stress with BMI demonstrated no significant correlation (r = -0.089, p = 0.381). Table 2 summarizes correlations between perceived stress scores and various clinical parameters.

**Table 2:** Correlation between perceived stress score and pulmonary function parameters. Pearson’s correlation was applied FEV1: Forced expiratory volume in 1 second, FVC: Forced vital capacity

Parameter	Correlation Coefficient (r)	p-value
Vital Capacity (L)	-0.1912	0.05
Forced Vital Capacity (L)	-0.1553	0.12
FEV1/FVC ratio (%)	0.0597	0.56

## Discussion

Our study examined psychological stress prevalence among individuals with COPD and explored potential gender differences in responses to this chronic respiratory condition. Our findings reveal a substantial psychological burden within the studied cohort, with approximately 74 (74%) of participants experiencing moderate to severe stress levels. This elevated prevalence underscores the significant psychological impact associated with COPD beyond its physiological manifestations.

The observed mean Perceived Stress Score (18.38 ± 7.41) aligns with findings from comparable research. For example, similar investigations have documented comparable psychological distress levels among COPD patients [15]. However, our findings indicate somewhat higher stress prevalence compared with studies conducted in more developed healthcare contexts, potentially reflecting differences in healthcare delivery systems, social support structures, or cultural factors influencing stress perception and reporting [16].

A particularly noteworthy discovery involves the substantial gender disparity in psychological stress, with female COPD patients reporting considerably higher stress levels compared to males (22.64 ± 5.36 vs. 17.85 ± 7.46, p = 0.04). This gender-based difference corresponds with existing literature examining psychological responses to chronic conditions. Previous research has similarly found that women with COPD experience elevated anxiety and psychological distress compared with men [17]. Multiple factors might contribute to this disparity, including differences in coping mechanisms, societal expectations, physiological stress responses, and potentially more limited social support networks for female patients [18].

The negative correlations between pulmonary function measurements and psychological stress suggest that physiological impairment may contribute somewhat to the psychological burden in COPD patients. Researchers have previously documented this relationship, finding declining respiratory function associated with increased psychological symptoms in COPD patients and medical students [19,20]. However, the relatively weak correlations in our investigation suggest that psychological stress in COPD likely stems from multiple sources beyond disease severity alone, potentially including psychosocial factors, coping resources, and individual resilience capacities [21]. Family support systems could significantly reduce stress levels in COPD patients, potentially improving both psychological well-being and disease management outcomes.

The absence of significant correlations between psychological stress and factors like age and BMI differs from some earlier studies. For instance, previous research has suggested associations between younger age and elevated psychological distress in COPD [22]. This discrepancy might reflect our sample’s relatively homogeneous age distribution or cultural factors specific to our study population.

Our findings carry important clinical implications. The high prevalence of psychological stress among COPD patients highlights the necessity for routine psychological assessment within comprehensive COPD management protocols. Furthermore, the observed gender differences suggest that gender-specific approaches to psychological support warrant consideration. Integrating stress management strategies, including mindfulness practices, cognitive behavioural therapy, and pulmonary rehabilitation programs addressing physical and psychological dimensions, could significantly enhance patient outcomes [23].

Several limitations merit consideration when interpreting these findings. First, the cross-sectional design precludes establishing causal relationships between COPD and psychological stress. Second, the relatively modest sample size, particularly the limited female participant representation, may affect the generalizability of gender-specific findings. Third, our investigation did not account for potential confounding variables such as socioeconomic status, educational background, and smoking history, which might influence psychological stress levels. Our single-center design may limit generalizability beyond similar tertiary care settings in Pakistan. Finally, while validated, the self-reported stress measurement approach may introduce reporting biases.

Future research directions should explore longitudinal relationships between disease progression and psychological well-being in COPD, investigate the effectiveness of gender-tailored psychological interventions, and examine how cultural and socioeconomic factors influence stress perception across different COPD populations. Additionally, more extensive studies with more balanced gender representation would provide more robust evidence regarding gender disparities in psychological responses to COPD.

By addressing both the physiological and psychological dimensions of COPD, healthcare providers can develop more comprehensive and effective management strategies that enhance the overall quality of life for this growing patient population.

## Conclusions

This study demonstrates that a substantial proportion of COPD patients experience moderate to severe psychological stress levels, with female patients reporting markedly higher stress compared to males. These findings emphasize the importance of incorporating routine psychological assessment within comprehensive COPD management protocols, with particular attention to gender-specific needs and responses. Our results highlight the need for targeted interventions addressing modifiable risk factors to improve COPD patients’ psychological health outcomes and overall quality of life. Healthcare policies and facilities for improving psychological health should be developed and employed, encompassing comprehensive screening programs, therapeutic interventions, and patient education initiatives.
